# Using a Neural Network to Improve the Optical Absorption in Halide Perovskite Layers Containing Core-Shells Silver Nanoparticles

**DOI:** 10.3390/nano9030437

**Published:** 2019-03-15

**Authors:** Michael D. Nelson, Marcel Di Vece

**Affiliations:** Interdisciplinary Centre for Nanostructured Materials and Interfaces (CIMaINa) and Physics Department “Aldo Pontremoli”, University of Milan, Via Celoria 16, 20133 Milan, Italy; michael.d.nels@gmail.com

**Keywords:** solar cell, plasmonics, light management, perovskite, core-shells, nanoparticles, silver 7, thin films, neural network, machine learning

## Abstract

Core-shells metallic nanoparticles have the advantage of possessing two plasmon resonances, one in the visible and one in the infrared part of the spectrum. This special property is used in this work to enhance the efficiency of thin film solar cells by improving the optical absorption at both wavelength ranges simultaneously by using a neural network. Although many thin-film solar cell compositions can benefit from such a design, in this work, different silver core-shell configurations were explored inside a Halide Perovskite (CH_3_NH_3_PbI_3_) thin film. Because the number of potential configurations is infinite, only a limited number of finite difference time domain (FDTD) simulations were performed. A neural network was then trained with the simulation results to find the core-shells configurations with optimal optical absorption across different wavelength ranges. This demonstrates that core-shells nanoparticles can make an important contribution to improving solar cell performance and that neural networks can be used to find optimal results in such nanophotonic systems.

## 1. Introduction

The efficiency improvement of commercial single junction solar cells, with active materials, such as silicon, CdTe and copper indium gallium selenide (CIGS), has been very gradual. This slow pace of solar cell efficiency improvement has led to the exploration of other compositions, such as organic, dye-sensitized copper zinc tin sulfide (CZTS) and Perovskite [1], which show fast increases in laboratory solar cell efficiency. Another approaches to increase the optical absorption in solar cells are light management techniques, such as those obtained by light scattering on Mie [2] or plasmonic [3] particles which randomizes the direction of light inside the solar cell and, thereby, increase the optical absorption probability [4]. The common methods, such as the use of simple plasmonic nanoparticles at various positions in diverse types of solar cells, have been studied extensively [5,6,7,8,9,10]. Although the lithographic design of plasmonic nanostructures provided valuable insight [11,12,13,14], its industrial use remains doubtful due to its costly method. Moreover, the Ohmic losses, i.e., the conversion of light into heat due to the electric resistance experienced by the oscillating electrons set in motion by the light, could hinder effective use of plasmonic scatterers. This concern has been addressed by a recent work by Disney et al. [15], in which it was shown that careful design of the plasmonic nanostructure results in a net increase of the solar cell efficiency.

To improve the plasmonic properties with respect to optical absorption in the surrounding semiconductor, such as Perovskite, the obvious parameters, such as size and shape, have been explored with little room for improvement [16]. In core-shells plasmonic-dielectric particles the optical response can be tuned further [17]. The plasmonic properties of nanostructures can be considered an equivalent electrical circuit where the metal sphere is approximated by a nano-inductor, nano-resistance, and nano “fringe” capacitor in parallel [18,19]. Furthermore, metal particles which consist of multiple dielectric-metal layers provide interesting parameters to improve the plasmonic performance, such as the number and thickness of shells. Due to the plasmonic coupling between the metal shell and the metal core, two plasmon resonances should occur; one broad plasmon resonance of the core and shell and one due to the coupling, which is located in the infrared [20]. This enables the simultaneous increase of optical absorption at different wavelength ranges. In a recent study by Peurifoy et al. [21] the scattering of such core-shells particles was optimized by using neural networks (NN). A neural network consists of a number of input parameters, an integrator with threshold determining functions which determine the output signals. The artificial neurons are connected to each other in hierarchical layers with the connections chosen as a training model. In a neural network, the dataset containing certain patterns, functions as input parameters, after which a feedback loop enhances the output result with respect to an optimized result by modifying the integrator settings. The neural network learns to produce a desired response to specific patterns and, therefore, can predict which patterns provide the optimum result, without being trained with that specific pattern. Neural networks are currently used in a wide range of applications and research [22,23], and although the successful use of neural networks in physics to extract patterns from large quantities of data has been published recently [24,25,26,27], its application to nanophotonics for solar cells is awaiting further developments. Because solar cells, and particularly complicated light management designs, have many free parameters, a neural network is ideal to find the optimum performance of the solar cell.

In this work, we present a study to improve the optical absorption in Halide Perovskite (CH_3_NH_3_PbI_3_) layers with plasmonic core-shells particles by training a neural network. Perovskite is a very promising solar cell material [28], which is being intensively investigated and improved [28,29,30,31]. Plasmonic nanostructures are feasible in principle [16,32,33,34] and will be the next advancement. Current laboratory Perovskite devices have an efficiency exceeding 20% towards 30%, which can be improved further in tandem solar cell configurations [35]. The replacement of organic compounds with inorganic compounds is an important way to improve stability and is a current topic of intensive research [36]. Self-healing of Perovskite solar cells may also contribute to its overall robustness [37] as well as defect-free perovskite films, and passivation of grain boundaries [38].

Plasmonic nanostructures in Perovskite solar cell devices are explored with simulations [16,32,33] and experiments [34], in which, for example, the inclusion of Au@TiO2 core−shell nanoparticles into porous TiO2 and/or perovskite semiconductor capping layers or gold nanoparticles at the bottom of the Perovskite layer led to efficiency improvements of about 40 to 50% [39,40]. Plasmonic nanostructures are also used to apply color to the Perovskite solar cell for esthetical reasons [41].

The optical properties of silver core-shells particles with a different number of shells and varying shell thickness were simulated by finite difference time domain (FDTD) simulations. The results were then used to train a neural network to find the optimal optical absorption in the Perovskite layer, which corresponds to a particular silver core-shells particle configuration. This work not only provides a design to plasmonically improve the efficiency of Perovskite solar cells but also provides a novel method to improve solar cell efficiency while making use of a neural network.

## 2. Methods

**Figure 1:**
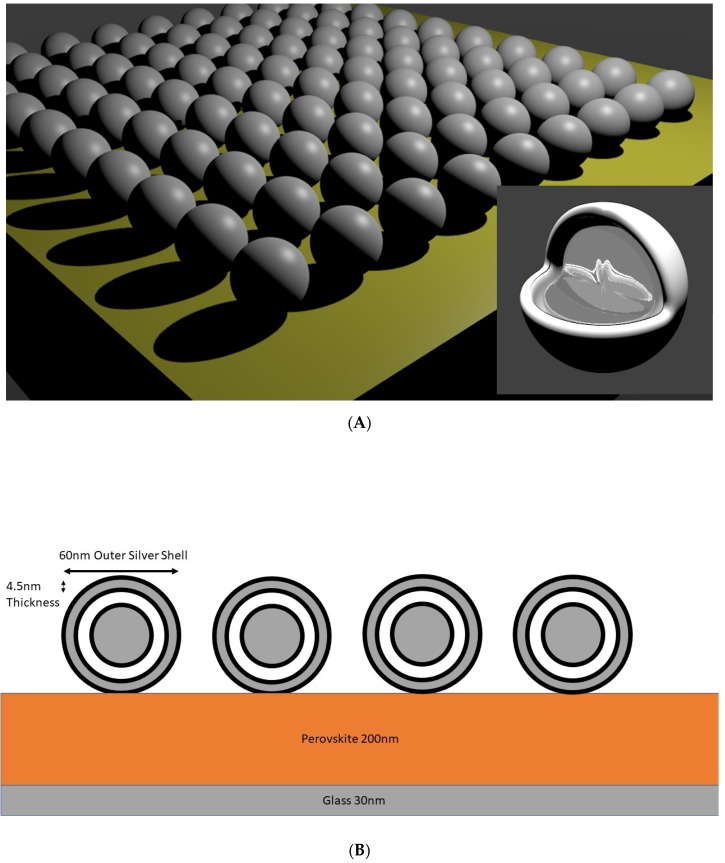
(**A**) An artistic depiction of one configuration with core-shells nanoparticles on top of a Perovskite thin film. The inset shows the different core and shells (glass and silver). (**B**) Detailed schematic illustration of the best performing core-shells setup.

The FDTD simulations were performed with commercial software (version 8.12, Lumerical Solutions Inc., Vancouver, BC, Canada) on core-shells particles using silver (Palik database [42]), with a varying radius of 20, 30, and 60 nm. An illustration and schematic depiction of the systems and setups are shown in Figure 1. The simulation box volume was 920 × 920 × 920 nm^3^ with 12 perfectly matched layers (PML) on each side. The silver core-shells particles were embedded on different positions in a 200 nm thick Halide Perovskite (CH_3_NH_3_PbI_3_ (MAPI)) [43] film, which was placed on top of a 30 nm thin glass plate. This small thickness was helpful to avoid interference effects leading to Fabry-Perot and guided photonic modes [11]. Two square power monitor boxes were placed around a single nanoparticle and around the silver nanoparticles array to monitor the optical absorption in Perovskite and silver. The monitors measured electric fields and dielectric properties, from which the optical absorption in each material was obtained. The plane light source was placed at a distance of 700 nm from the bottom of the perovskite layer with a spectral range of 300 to 1200 nm. The simulation optical absorption in Perovskite was adjusted to compensate for the volume lost by the particle array.

The neural network was created by commercial software (Neural Designer, 3.0, Salamanca Spain), and three models were used for the nanoparticle array. The scaling layers were always kept at automatic, the principal components layer was not used while the unscaling and bounding layers alternated between either off or on with minimum/maximum methods. The number of layers and the number of neurons in each layer differed for each model. The activation function for each layer was always the hyperbolic tangent, while the output layer always had a linear activation function. The loss index error method was the only variable that changed in the training strategy, while the order selection and input selection remained the same for each model. Three neural network models for the array were explored (3371, 3311, 33341, each number indicating the number of neurons in the layer) with an average error of maximum of 2.5%. Each neural network model was trained once the network parameters were set, after which order selection was run to minimize the loss on the selection instances. This process was repeated twice, and after each iteration, the network automatically updated the network parameters to give the best results. The neural network code was then further analyzed to find the configurations given by the neural network with the highest absorption for each wavelength value in the three ranges and to find the configuration with the highest average absorption.

Although an infinite number of neural network models could be investigated, here we only present a feasibility study in which three models are sufficient. The simulation parameters that were changed included the particle size (20, 30, and 60 nm), number of shells (maximum of three metal shells), and silver shell thickness (1.5, 2.25, 3, 4, and 4.5 nm) and the position of the particle within/on the Perovskite layer (20, 40, 95, 160, 180, 210, 215, and 230 nm from the glass upper surface). The detailed combinations of realized simulations are listed in the Supplementary Material. The intermediate layers between the silver shells consisted of a glass shell. The core and outer layer of the particles could be either silver or glass.

## 3. Results and Discussion

**Figure 2:**
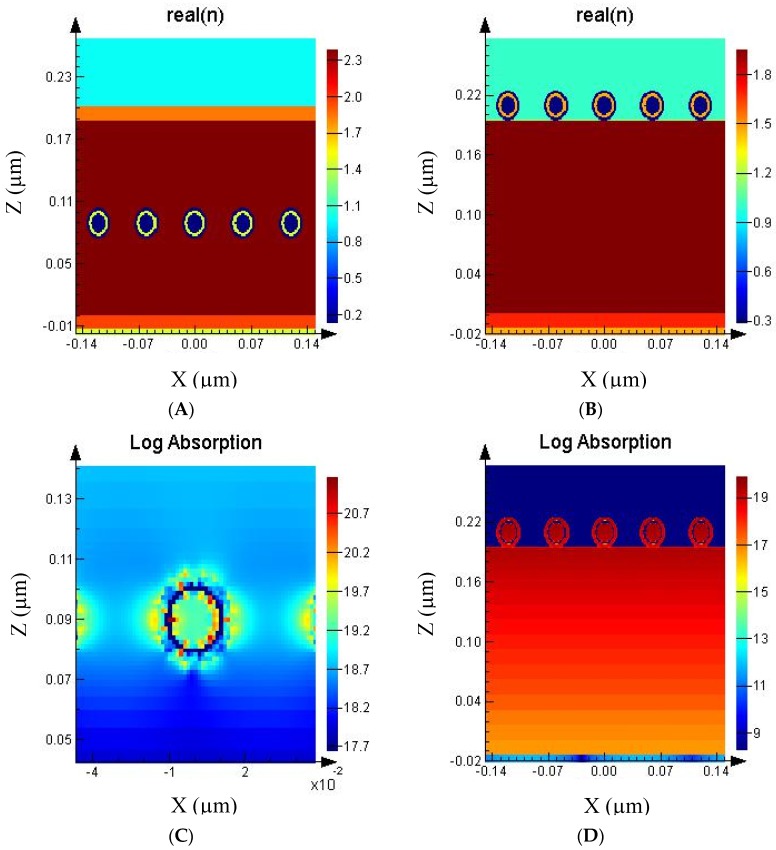
Refractive index (n) of the silver core-shells particle (30 nm) array (**A**) inside Perovskite and (**B**) on top of the Perovskite thin film. The right color bar indicates the real n value. The red corresponds to Perovskite, blue with silver and yellow with glass. (**C**) The log of the optical absorption through the x-z plane (parallel to light direction) of a single core-shells particle inside Perovskite and (**D**) through the x-z plane (perpendicular to light direction). The origin of the monitor boxes was placed at the bottom of the Perovskite layer which results in negative position values for the glass layer. The intensity scale bar units are log absorption (ratio incident power and absorbed power) m-3. The cross sections are recorded at 555 nm for the particles inside Perovskite and 336 nm for the nanoparticle on top of the Perovskite thin film, which corresponds to the first silver plasmon resonance peak.

The illustrations of Figure 1A,B show a configuration with the core-shells nanoparticles on top of the Perovskite thin film, with the inset showing a cross section of the core and shells of this particular configuration. The optical effect of the core-shells particles can be determined by measuring the optical absorption in and around them as shown in the example in Figure 2. The chosen configuration of the core shell particle is shown by the refractive index cross-section on the array with the core-shell nanoparticles inside (A) and on top (B) of the Perovskite thin film. In Figure 2C the optical absorption is shown parallel to the incident light and its polarization direction (electric field component E) at a position inside the Perovskite layer where a stronger absorption is visible closer to the surface. The blue ring around the silver core particle indicates minimal optical absorption in the glass shell, while the silver core and outer silver shell absorb much more light. Close to the outer shell and inside the Perovskite, the strong absorption is due to local field enhancement [44,45,46], which contributes to the over-all optical absorption from the particle. The optical absorption is greatest between the particles in the direction parallel to the light polarization (E-field) due to the dipole nature of the plasmonic response [47]. The higher optical absorption between the particles can also indicate a low strength plasmonic coupling [20] of the particles due to the dipole nature of the plasmonic resonance. When the core-shells particles are placed on top of the Perovskite layer (Figure 2D) it is clear that no strong field enhancement is present and that, therefore, the effect of the core-shells nanoparticles can only be the results of scattering. Nanoparticle scattering can occur in many different directions making an optical absorption pattern difficult to observe. The absence of optical absorption patterns in the cross sections indicates that the periodic arrangement results only in minimal resonance effects. Moreover, the very thin Perovskite layer does not support interference at the wavelengths measured here.

**Figure 3:**
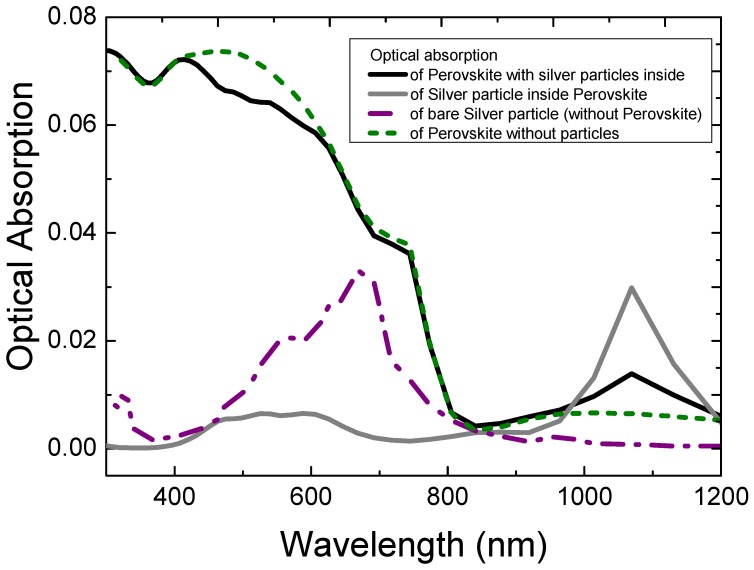
Optical absorption spectra of Perovskite with and without silver core-shell particle and of silver with and without Perovskite layer (surrounding). The silver nanoparticle had a silver core diameter of 20 nm and a silver shell of 3 nm thickness with glass in between. The outer diameter of the particles was 30 nm.

The optical absorption spectra in Perovskite with and without silver core-shells particles are shown in Figure 3 along with a silver core-shells particle with and without Perovskite. The optical absorption of bare silver particles has an initial peak at around 550 nm, which is the red-shifted silver particle plasmon peak, due to the surrounding Perovskite with a high refractive index. The surrounding Perovskite optical absorption is slightly reduced around this wavelength as compared with the Perovskite without nanoparticles, due to the antenna properties of the silver core-shells nanoparticle [3,48], which directs radiation to the particle and converts it into heat by collective electron motion. At around 1100 nm the optical absorption in Perovskite and the silver particle have increased substantially as compared with their bare counterparts. The increased absorption in silver indicates a second silver particle plasmon resonance, due to the coupling between the metallic core and metallic shell, which is considerably red-shifted due to the extended length [49]. Because the optical absorption in Perovskite at this wavelength is much smaller compared to around 555 nm (the first plasmon resonance peak), the plasmon response is able to produce a net optical absorption gain. For each different silver core-shells particle configuration, a trade-off between optical absorption in Perovskite and silver at different wavelengths produces a net optical absorption gain or loss.

In a solar cell, only the optical absorption of the active layer determines the light conversion efficiency. Because of this, the optical absorption spectra of Perovskite, containing the various core-shell silver particles at the different positions in the Perovskite layer, were used to train the neural network. Three different wavelength regions were used to investigate the neural network models, 500–600 nm, which is above the absorption edge, 700–800 nm which is on the absorption edge and 1000–1200, which is in the infrared, outside the absorption edge.

**Figure 4:**
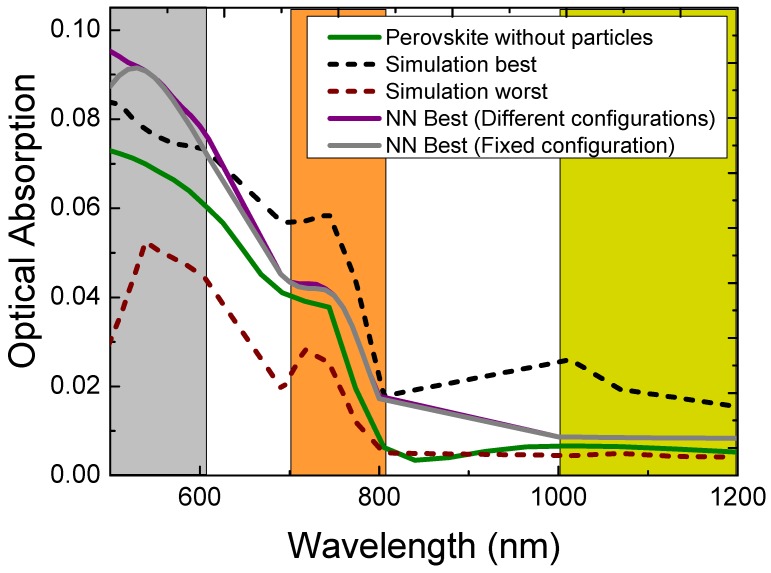
Optical absorption spectra of Perovskite without silver core-shell particle, with core-shell nanoparticles with best and worst simulated configuration, the neural network best results with fixed and varying configurations. The colored bands indicate the three wavelength ranges used to analyze the neural network results.

In Figure 4, the optical absorption is shown for the best and worst results of the simulation, which varies very strongly around the normal absorption in Perovskite. It is clear that the core-shell nanoparticles attract electromagnetic radiation due to their high optical density and either absorb or re-radiate the light. Re-radiation can occur by scattering or local field enhancement of the dipole response. The detrimental effect of the core-shell nanoparticles on the optical absorption in Perovskite can be about 50%, while the improvement can be about 30%. The best core-shells particles were mainly positioned at the top of the Perovskite layer with three metallic shells (3 nm thick), a metallic core and outer shell with a diameter of 60 nm for the range of 500 to 600 nm. For the range, 700 to 800 nm the core-shells particles were positioned inside the Perovskite layer with one or two metallic shells of 4 or 4.5 nm thickness, with mostly a metallic core and oxide shell with an outer diameter of 60 nm. For the range, 1000 to 1200 nm the core-shells particles were positioned at the bottom of the Perovskite layer, with a varying number of shells of mostly 3 nm thickness. The core-shells particles always had a metallic core, while the outer shell varied between metallic and oxide, with a varying outer diameter between 20 and 60 nm. The worst simulation results in the 500 to 600-nm range often had core-shells particles positioned inside the Perovskite layer or had about two metallic shells when they were on top of the Perovskite layer. Although the diameter of 60 nm did not change, the outer shell was oxide for the particles on top of Perovskite. In the range, 700 to 800 nm the core-shells particles were positioned closer to the top surface, while the number of metallic shells was either one or three, the diameter was sometimes 30 nm, and the core and outer shell varied between metallic and oxide. For the 1000 to 1200 nm range, the worst performing core-shells particles were positioned on the top of the Perovskite layer with mostly 3 metallic shells with the outer shell metallic and a diameter of 60 nm.

The neural network models were analyzed for two situations: the highest optical absorption in Perovskite with: (1) allowing a different configuration for each wavelength, and (2) the overall best configuration for all the wavelengths in the relevant wavelength range. The optical absorption curves of these two neural network results are also shown in Figure 4 and are mostly overlapping in the measured wavelength range. The neural network best results are not always better than the simulation best results because the optical absorption for the two cases depends on the wavelength. For both cases, the neural network is only able to improve the optical absorption in Perovskite around 500 nm. This improvement was possible with one model of the three used neural network models, which was then used for Figure 4. In this wavelength range, the most beneficial position was 225 nm, with only one metallic shell of thickness 1.5 nm, an outer diameter of 60 nm, a silver core and silver outer shell. For a few wavelengths (580–600 nm) a shell thickness of 4.5 nm performed better in the neural network best results. Remarkably, this particular configuration (diameter 60 nm, silver core and silver outer shell with 4.5 nm thickness) was not simulated and, therefore, a prediction of the neural network. The position of the core-shells particle at 225 nm, at the top of the Perovskite film, indicates that scattering of light is the best approach to increase the optical absorption in Perovskite with photon energies larger than that of the band gap. Furthermore, the size of 60 nm, which was the maximum diameter used in the simulations is in agreement with stronger scattering of nanoparticles when the size increases [50,51]. The neural network was only able to perform better than the simulation around 500 nm because the silver plasmon resonance wavelength is around that wavelength. At the plasmon resonance, scattering is strongest and, therefore, provides a “handle” for the neural network to optimize. With one metallic shell around a core, the oscillation strength was increased due to the plasmonic coupling between the core and shell, increasing its scattering power, while more shells did not increase the scattering power.

Although the simulations in the wavelength range 1000 to 1200 nm provided increased optical absorption with core-shell particles included at the bottom of the layer, the neural network was not able to optimize this and did not even outperform the simulation. Although a plasmon resonance occurs in this wavelength range for some core-shell nanoparticles, the neural network was not able to find a pattern due to unpredictable properties of the various core-shells nanoparticle configurations. While both the best simulations and the neural network placed the core-shells nanoparticles at the bottom, the best simulations had a diameter of 30 nm with a shell thickness of mostly 3 nm, while the neural network resulted in particles with a diameter of 60 nm with a shell thickness of 4 nm. In this wavelength range Perovskite does not absorb much light, therefore, most of the incident light reaches the bottom where a nanostructure can scatter it back into the active layer. The maximum scattering power is also shifting as a function of size because the plasmon resonance wavelength of the nanoparticles inside the Perovskite depends on their size. Because only a limited wavelength range (1000–1200 nm) was used to train the neural network, the plasmon resonance wavelength can move outside this wavelength window, complicating the optimization. Interestingly, the nanoparticles which performed best in this wavelength range as obtained from the simulations and neural network almost always had a glass shell around the silver shell to optimize the plasmon resonance. Although this is a proof of principle study, modern deposition techniques will be able to manufacture such complicated systems by, for example, approximating core-shells particles by layered deposition on top of simple particles. Although for some Perovskites the formation of AgI_2_ due to the presence of iodine cannot be excluded [52,53], the formation of protective oxide layers or using different metals could contribute to robust core-shells for photovoltaics. Another complication is the enhanced electron-hole recombination at the metal-semiconductor (Perovskite) interface, which could be countered by an oxide layer between the metal and semiconductor.

## 4. Conclusions

Core-shells nanoparticles have various “handles” to control the plasmon resonance wavelength and strength, such as the number or thickness of the shells. Some core-shells nanoparticles have two plasmon resonances which can be conveniently tuned to the wavelength region of interest with respect to the semiconductor of a solar cell. Both the FDTD simulations and the neural network showed that core-shells nanoparticles increase the optical absorption in Perovskite thin films when they are placed on top for photon energy comparable to the band-gap and at the bottom of the Perovskite thin film for photon energies smaller than the band gap. The neural network used was able to find an optimal configuration of the core-shells nanoparticles which was not simulated. This demonstrates the potential of neural networks for nanophotonics and improving solar cell efficiency with intricate light management nanostructures. Future research using neural networks for solar cells could focus on different shapes of particles, i.e., cubes, stars etc., or different compositions, such as gold or aluminium. Because of the complex structure of Perovskites, neural networks could be used to find optimized and more stable compositions.

## Supplementary Materials

The following are available online at https://www.mdpi.com/2079-4991/9/3/437/s1, Table S1: Simulation Configuratations.
