# Supplementary material for: Using a Neural Network to Improve the Optical Absorption in Halide Perovskite Layers Containing Core-Shells Silver Nanoparticles
Source: Nanomaterials (Basel). 2019 Mar 15;9(3):437. doi: 10.3390/nano9030437 (PMC6474077; doi:10.3390/nano9030437)
Supplement: Supplementary file 1 [file nanomaterials-09-00437-s001.pdf]

Supplementary Material:
Using a neural network to improve the efficiency of Perovskite solar cells containing core-shells silver nanoparticles. Michael D. Nelson and Marcel Di Vece
Interdisciplinary Centre for Nanostructured Materials and Interfaces (CIMaINa) and Physics Department "Aldo Pontremoli", University of Milan, Via Celoria 16, 20133, Milan, Italy
Simulation configurations:

Table 1. Simulation Configuratations.

Run #	Metallic Layers	Thickness (nm)	Outer Diameter (nm)	Silver Center (1=yes, 0=no)	Silver Outer (1=yes, 0=no)	Layering (S=Ag, G=Glass)	Positions from bottom of Perovskite layer (nm)	Particle Array
1	2	1.5	20	1	0	SGSG	15, 90, 175, 205	14 x 14
2	3	2.25	30	1	1	SGSGS	15, 90, 175, 210	14 x 14
3	3	1.5	30	1	1	SGSGS	15, 90, 175, 210	14 x 14
4	3	3	60	1	1	SGSGS	35, 90, 155, 225	9 x 9
5	2	4.5	60	1	0	SGSG	35, 90, 155, 225	9 x 9
6	1	1.5	20	1	1	S	15, 90, 175, 205	14 x 14
7	1	1.5	20	1	0	SG	15, 90, 175, 205	14 x 14
8	1	3	30	1	0	SG	15, 90, 175, 210	14 x 14
9	1	1.5	20	0	1	GS	15, 90, 175, 205	14 x 14
10	1	3	30	0	1	GS	15, 90, 175, 210	14 x 14
11	2	1.5	20	1	1	SGS	15, 90, 175, 205	14 x 14
12	2	3	30	1	1	SGS	15, 90, 175, 210	14 x 14
13	1	1.5	20	0	0	GSG	15, 90, 175, 205	14 x 14
14	1	3	30	0	0	GSG	15, 90, 175, 210	14x14
15	2	3	30	1	0	SGSG	15, 90, 175, 210	14 x 14
16	3	1.5	20	1	1	SGSGS	15, 90, 175, 205	14 x 14
17	3	3	30	1	1	SGSGS	15, 90, 175, 210	14 x 14
18	3	4	60	1	1	SGSGS	35, 90, 155, 225	9 x 9
19	1	4	60	0	0	GSG	35, 90, 155, 225	9 x 9
